# Copy number variation and cytidine analogue cytotoxicity: A genome-wide association approach

**DOI:** 10.1186/1471-2164-11-357

**Published:** 2010-06-04

**Authors:** Krishna R Kalari, Scott J Hebbring, High Seng Chai, Liang Li, Jean-Pierre A Kocher, Liewei Wang, Richard M Weinshilboum

**Affiliations:** 1 Division of Biostatistics and Informatics, Department of Health Sciences Research, Mayo Clinic, Rochester, MN 55905, USA; 2 Division of Clinical Pharmacology, Department of Molecular Pharmacology and Experimental Therapeutics, Mayo Clinic, Rochester, MN 55905, USA

## Abstract

**Background:**

The human genome displays extensive copy-number variation (CNV). Recent discoveries have shown that large segments of DNA, ranging in size from hundreds to thousands of nucleotides, are either deleted or duplicated. This CNV may encompass genes, leading to a change in phenotype, including drug response phenotypes. Gemcitabine and 1-β-D-arabinofuranosylcytosine (AraC) are cytidine analogues used to treat a variety of cancers. Previous studies have shown that genetic variation may influence response to these drugs. In the present study, we set out to test the hypothesis that variation in copy number might contribute to variation in cytidine analogue response phenotypes.

**Results:**

We used a cell-based model system consisting of 197 ethnically-defined lymphoblastoid cell lines for which genome-wide SNP data were obtained using Illumina 550 and 650 K SNP arrays to study cytidine analogue cytotoxicity. 775 CNVs with allele frequencies > 1% were identified in 102 regions across the genome. 87/102 of these loci overlapped with previously identified regions of CNV. Association of CNVs with gemcitabine and AraC IC_50 _values identified 11 regions with permutation p-values < 0.05. Multiplex ligation-dependent probe amplification assays were performed to verify the 11 CNV regions that were associated with this phenotype; with false positive and false negative rates for the in-silico findings of 1.3% and 0.04%, respectively. We also had basal mRNA expression array data for these same 197 cell lines, which allowed us to quantify mRNA expression for 41 probesets in or near the CNV regions identified. We found that 7 of those 41 genes were highly expressed in our lymphoblastoid cell lines, and one of the seven genes (*SMYD3*) that was significant in the CNV association study was selected for further functional experiments. Those studies showed that knockdown of *SMYD3*, in pancreatic cancer cell lines increased gemcitabine and AraC resistance during cytotoxicity assay, consistent with the results of the association analysis.

**Conclusions:**

These results suggest that CNVs may play a role in variation in cytidine analogue effect. Therefore, association studies of CNVs with drug response phenotypes in cell-based model systems, when paired with functional characterization, might help to identify CNV that contributes to variation in drug response.

## Background

It is known that inherited genomic CNV is linked to risk for human disease and response to treatment. It has also been established for decades that genomic variation, including CNV in germline DNA, can help predict variation in efficacy and/or adverse responses to therapeutic drugs [1-5]. For example, individuals with multiple copies of the gene encoding the drug metabolizing enzyme CYP2D6 are "ultrarapid" metabolizers as compared to those with *CYP2D6 *deletions ("poor" metabolizers), and these genotypes are associated with variation in response to a large number of drugs [4,6]. CNVs within the human genome are not rare events. Redon et. al. [7] identified nearly 1,500 CNV regions scattered throughout the genome in 270 HapMap samples. Those regions comprised approximately 10% of the human genome, encompassing coding and non-coding regions, as compared to the < 1% of the genome that is occupied by SNPs [8]. CNVs appear to be present at lower frequencies than SNPs [9], but this may be due in part to the techniques utilized to identify them. Thus, the prevalence and biological significance of CNVs may be underestimated. As of early 2009, nearly 6,225 CNV loci had been cataloged by the Database of Genomic Variants http://projects.tcag.ca/variation/. In addition, nearly 18% of mRNA species that are genetically regulated through *cis *effects could be explained by CNVs [10]. Together with SNP genotypes, CNV data can be generated with SNP arrays [7,9,11-13]. Although these methodologies have limitations [14], CNVs, depending on their size and location, may be just as important for variation in function as are SNPs.

The cytidine analogues, gemcitabine and AraC, show significant therapeutic effect in several types of cancer. Gemcitabine is mainly used to treat solid tumors [15,16] while AraC is used to treat acute myelogenous leukemia [17]. Clinical response to these two drugs varies widely, and previous studies showed that inheritance can contribute to the variation in response of these two drugs [18]. In this study, we set out to test the hypothesis that CNV might contribute to variation in gemcitabine and AraC response in 197 EBV transformed lymphoblastoid cell lines using SNP data obtained with Illumina 550 and 650 K SNP arrays.

## Methods

### Genotyping and populations

A subset of the "Human Variation Panel" lymphoblastoid cell lines consisting of 60 Caucasian-American (CA), 54 African-American (AA), and 60 Han Chinese-American (HCA), as well as 23 CEPH Caucasian HapMap EBV transformed cell lines was obtained from the Coriell Cell Repository (Camden, NJ). These cell lines had been obtained from healthy individuals and were anonymized by the National Institute of General Medical Sciences prior to deposit. All of these individuals had provided written consent for the use of their cells and DNA from those cells to be used for experimental purposes. We genotyped the AA DNA from these cell lines using the Illumina Human Hap 650 beadchip (Human660W-Quad v1), and the Illumina Human Hap 550 beadchips were used to genotype the remainder of the samples. All samples were genotyped in the Mayo Clinic Genotyping Core Facility. All but two samples had a call rate greater than 98%, and those two samples, even after repetition, had call rates between 95 and 98%. We assessed LRR standard deviation (SD) for our samples and found that none of the samples had a SD less than 0.21. Quality control (QC) recommendations for the PennCNV or QuantiSNP algorithms suggest using a SD < 0.3 [19]. Since, our LRR standard deviation did not exceed this QC threshold; we also used those two samples in the analysis. For consistency, we did not include the additional 100 K SNPs genotyped for the AA samples in the CNV analysis.

### Copy number identification

Bead Studio version 3.1 was used to obtain log R ratios and B allele frequencies for 550,000 SNPs in the 197 samples studied. LogR ratios were generated by comparing our experimental LogR values to Bead Studio's built in multi-ethnic HapMap population. CNV genotyping was performed using an Objective Bayes Hidden-Markov model (QuantiSNP) [20] plug-in within Illumina's Bead Studio interface. QuantiSNP is a statistical algorithm that utilizes joint information with regard to log R ratios and B allele frequencies for quantitative SNP array data analysis that allows for precise discovery and mapping of copy number changes. We used the QuantiSNP parameters recommended by Illumina: expectation maximization = 10, CNV length = 10,000, maximum copy number returned = 4, no GC content normalization, and score threshold = 50. After applying the QuantiSNP algorithm, we exported the CNV values and confidence values for each SNP out of the Bead Studio software. Using our own in-house programs written in R and Perl, we then separated all SNPs associated with CNVs that were observed in two or more samples (frequency > 1%). These thresholds and parameters were set conservatively to accurately identify CNVs under these conditions. This approach should reduce the false positive rate, but at the risk of increasing the false negative rate and missing more common, yet smaller CNVs that are inherently more difficult to detect. After transforming CNV values for each SNP into deletion (CNV value < 2), normal (CNV value = 2) or amplification (CNV value > 2), distinct copy number regions were obtained by merging neighboring SNPs with identical CNVs across samples. A detailed description of the methods used is available in the Additional file 1 Methods Section.

### Copy number validation

Eleven copy number regions found to be associated with gemcitabine and AraC IC_50 _values (p < 0.05) were selected for validation using multiplex ligation-dependent probe amplification (MLPA). Oligonucleotides were preferentially designed based on a successful assay, followed by selection for coding sequences and underlying p-values. M13 sequence was attached to each probe together with a complementary FlexMap100 sequence (Luminex, Austin, TX). Specifically, 80 ng of DNA was denatured at 98°C for 5 minutes, followed by 25°C for 1 minute. In an 8 μL reaction, 80 ng of DNA and 0.3 femtomole/μL of each probe were mixed with 1.5 μL of MLPA buffer (MRC-Holland, Amsterdam, Netherlands). Probes were allowed to hybridize at 60°C for 16-24 hours. Probes were ligated in a reaction containing 25 μl H_2_O, 3 μL Ligase-65 Buffer A and B (MRC-Holland), and 1 μL Ligase-65 (MRC-Holland) at 54°C for 15 minutes, followed by 98°C for 5 minutes. Each 50 μL PCR reaction consisted of 10 μL of ligated product mixed with 27.5 μL H_2_O, 5 μL 10 × buffer (Invitrogen, Carlsbad, CA), 1.5 μL 50 mM MgCl_2 _(Invitrogen), 4 μL 10 mM dNTPs (Applied Biosystems, Foster City, CA), 0.5 μL 10 μM M13 primers, and 1 μL Platinum Taq (Invitrogen). 10 μL of PCR product was then added to 40 μL of bead mix containing 2,000 beads for each FlexMap Microsphere (Luminex) suspended in 1 × TMAC, and the mixture was incubated at 96°C for 2 minutes, followed by 37°C for 60 minutes. Following incubation, 0.2 μL of Streptavidin R-Phycoerythrin Conjugate (Invitrogen) plus 25 μL of 1 × TMAC was added and incubated at room temperature for 30 minutes. Samples were assayed on a LiquiChip 100IS System (Qiagen, Valencia, CA) and results were analyzed with GeneMarker 1.6 software. Of the 11 CNV assayed, one (chr14CNV87:106047919-106066496), did not provide adequate signal intensity for analysis.

### MTS assay

AraC was purchased from Sigma-Aldrich (St. Louis, MO) and gemcitabine was provided by Eli Lilly (Indianapolis, IN). Cytotoxicity assays were performed with the CellTiter 96^® ^Aqueous Non-Radioactive Cell Proliferation Assay (Promega Corporation, Madison, WI). The drug concentrations used to perform these experiments were described in detail previously by Li et al., 2008 [18].

### Statistical analysis

The cytotoxicity phenotype (IC_50_) was determined on the basis of the best fitting curve, either 4 parameter logistic, 4 parameter logistic with top = 100%, or 4 parameter logistic with bottom = 0%. The curves were constructed using the dose response curves package in R. The logistic model with the lowest mean square error was used to determine IC_50 _values for gemcitabine and AraC as described in Li et al. [18]. Drug response phenotypes (IC_50 _values) for both drugs were adjusted for ethnicity, gender and storage time of the 197 samples using linear regression (natural log transformation applied to IC_50 _values). In addition, CNV values were adjusted for ethnicity and gender. Linear regression was then used to perform association with adjusted CNV values (residuals from regressing CNV against ethnicity and gender) with adjusted IC_50 _phenotypes (residuals from regressing log IC_50 _against ethnicity, gender and storage time). P-values for association were obtained after performing 1000 permutations for both gemcitabine and AraC IC_50 _values.

### Transient transfection and RNA interference

Human MiaPaca-2 pancreatic cancer cells were transfected with siRNA using Lipofectamine RNAMAX (Invitrogen). Specifically, cells were seeded into 96-well plates and were mixed with siRNA-complex containing 50 nM specific or negative control siRNA (Qiagen) and transfection reagent (Invitrogen) in Opti-MEM^® ^I Reduced Serum Media (Invitrogen). Forty eight hours post-transfection, cells were harvested for cell-based assays. SMYD3 siRNA and negative control siRNA were purchased from Qiagen and were used as suggested by the manufacturer.

Sequences for siRNA against SMYD3 were: Sense strand: GGC GAU CAU AAG CAG CAA UdTdT CGA UUA UAA UAA AUU CAA CdTdT

Antisense strand: AUU GCU GCU UAU GAU CGC CdTdT UUU GAA UUU AUU AUA AUC GdTdG

Sequences for negative control siRNA were:

Sense strand: UUC UCC GAA CGU GUC ACG UdTdT

Antisense strand: ACG UGA CAC GUU CGG AGA AdTdT

### Real-time quantitative reverse transcription-PCR

Total RNA was isolated from cultured cells with the Qiagen RNeasy kit (Qiagen), followed by QRT-PCR performed with the 1-step, Brilliant II SYBR Green QRT-PCR master mix kit (Stratagene, La Jolla, CA). Specifically, primers purchased from Qiagen were used to perform QRT-PCR using the Stratagene Mx3005P™ Real-Time PCR detection system (Stratagene). All experiments were performed in triplicate with β-actin as an internal control. Control reactions lacked RNA template.

## Results

### CNV identification

**Figure 1:**
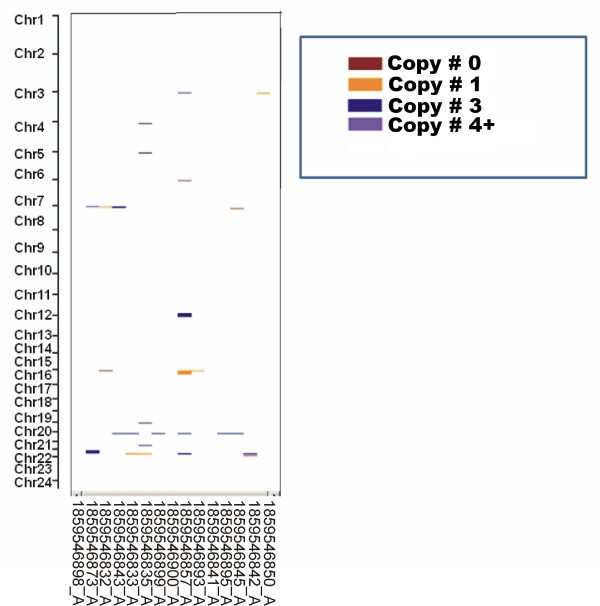
**Visualization of copy number regions identified in 15 randomly selected samples using Bead studio software**. Randomly selected individual samples are listed on the X-axis and chromosomes on the Y-axis. Each colored bar represents one CNV call. Colors indicate copy number; where dark red indicates copy # 0, dark orange indicates copy # 1, dark blue indicates copy # 3, blue violet indicates copy # 4+. The thickness of the band indicates the length of the CNV region.

We used the QuantiSNP parameters recommended by Illumina for copy number identification. The QuantiSNP algorithm in Illumina provided CNV values and confidence values for each SNP and sample. After pre-processing the data, we had 73,738 SNPs with CNV values other than "normal" (CNV value = 2). 1,674 SNPs were retained in the analysis after excluding SNPs that did not display variation in at least two samples (minor allele frequency > 1%). We then applied a simple segregation algorithm as described in Additional file 1 Methods and identified 775 CNVs at 102 loci using the 197 DNA samples obtained from 3 ethnic groups. Figure 1 shows the CNV call results using CNV region display in Bead Studio Software for 15 samples selected randomly from among the 197 samples assayed. From the randomly selected data displayed in Figure 1, it is clear that specific CNV regions could be associated with multiple DNA samples. Copy number loci or regions can also have multiple forms of variation, probably as a result of different breakpoints. The mean and median CNV frequencies per sample were 3.9 and 4.0, respectively, with a maximum value of 10. The 102 CNV loci identified represented 7.8 Mb of sequence, with an average length of 77 kb and a median of 20 kb (90 bp to 1.7 Mb) (Table 1). Twenty five CNVs identified in this study were observed in all three ethnic groups; 45 CNVs were found in 2 ethnicities; and 32 CNVs were observed in only one ethnic group (Table 1). No loci below 1% copy number frequency were reported in this study.

**Table 1:** CNV regions identified in 197 DNA samples.

CNV_ID	Start	Stop	Size	AA	CA	HCA	Combined CNV Frequency
chr1CNV1	12789177	12834675	45498	0.036	0.024	0.050	0.035

chr1CNV2	94906770	94925850	19080	0.091	0.000	0.000	0.025

chr1CNV3	105966892	106000090	33198	0.055	0.000	0.017	0.020

chr1CNV4	147306690	147414362	107672	0.036	0.012	0.000	0.015

chr1CNV5	187665261	187809352	144091	0.000	0.000	0.083	0.025

chr1CNV6	195092486	195160949	68463	0.073	0.000	0.083	0.045

chr1CNV7	243707190	243713984	6794	0.000	0.036	0.000	0.015

chr2CNV8	41092376	41099005	6629	0.273	0.024	0.017	0.091

chr2CNV9	57281457	57295357	13900	0.000	0.036	0.000	0.015

chr2CNV10	89397452	89877778	480326	0.036	0.060	0.017	0.040

chr2CNV11	110228954	110315618	86664	0.036	0.012	0.050	0.030

chr2CNV12	184808081	184866619	58538	0.127	0.012	0.000	0.040

chr2CNV13	242566407	242653950	87543	0.000	0.084	0.000	0.035

chr3CNV14	6194326	6211038	16712	0.073	0.000	0.000	0.020

chr3CNV15	53003415	53010084	6669	0.000	0.048	0.017	0.025

chr3CNV16	65168286	65187636	19350	0.018	0.120	0.117	0.091

chr3CNV17	75535790	75610832	75042	0.000	0.036	0.000	0.015

chr3CNV18	101837214	101854561	17347	0.000	0.036	0.000	0.015

chr3CNV19	152997395	153028291	30896	0.000	0.036	0.000	0.015

chr3CNV20	163614102	163617940	3838	0.018	0.060	0.000	0.030

chr3CNV21	163701543	163710564	9021	0.000	0.012	0.283	0.091

chr3CNV22	166750382	166766442	16060	0.036	0.012	0.000	0.015

chr3CNV23	177371924	177397828	25904	0.000	0.072	0.000	0.030

chr3CNV24	189070613	189088009	17396	0.000	0.012	0.033	0.015

chr3CNV25	192548615	192550982	2367	0.000	0.120	0.000	0.051

chr4CNV26	64386888	64391664	4776	0.000	0.024	0.033	0.020

chr4CNV27	64780488	64795145	14657	0.000	0.000	0.050	0.015

chr4CNV28	88405746	88445557	39811	0.055	0.000	0.000	0.015

chr4CNV29	104433739	104454829	21090	0.091	0.000	0.000	0.025

chr4CNV30	161277505	161290832	13327	0.000	0.060	0.000	0.025

chr4CNV31	162083578	162175279	91701	0.000	0.012	0.083	0.030

chr5CNV32	9955403	9976731	21328	0.036	0.036	0.000	0.025

chr5CNV33	97075236	97107276	32040	0.036	0.036	0.000	0.025

chr5CNV34	117418457	117420311	1854	0.109	0.060	0.000	0.056

chr5CNV35	120337992	120440119	102127	0.055	0.000	0.000	0.015

chr5CNV36	160474752	160479606	4854	0.000	0.000	0.050	0.015

chr2CNV37	67076651	67104015	27364	0.018	0.181	0.000	0.081

chr6CNV38	79031111	79086086	54975	0.255	0.410	0.083	0.268

chr6CNV39	93632051	93634213	2162	0.073	0.000	0.017	0.025

chr6CNV40	167619368	167688151	68783	0.055	0.000	0.000	0.015

chr7CNV41	64334979	64553672	218693	0.000	0.024	0.033	0.020

chr7CNV42	76038186	76394983	356797	0.073	0.036	0.033	0.045

chr7CNV43	81761377	81761849	472	0.000	0.000	0.050	0.015

chr7CNV44	89187436	89247424	59988	0.036	0.012	0.000	0.015

chr7CNV45	141420759	141433796	13037	0.036	0.060	0.067	0.056

chr8CNV46	3775146	3776955	1809	0.055	0.036	0.050	0.045

chr8CNV47	3987675	3990899	3224	0.073	0.000	0.000	0.020

chr8CNV48	5583294	5591685	8391	0.018	0.036	0.000	0.020

chr8CNV49	13643904	13652680	8776	0.036	0.012	0.000	0.015

chr8CNV50	13657871	13680110	22239	0.182	0.012	0.000	0.056

chr8CNV51	16307052	16309085	2033	0.018	0.048	0.000	0.025

chr8CNV52	72378670	72378984	314	0.000	0.036	0.000	0.015

chr8CNV53	137757412	137919630	162218	0.018	0.036	0.000	0.020

chr9CNV54	507715	513495	5780	0.055	0.000	0.000	0.015

chr9CNV55	581094	597738	16644	0.073	0.000	0.000	0.020

chr9CNV56	5376384	5382199	5815	0.109	0.000	0.000	0.030

chr9CNV57	9782326	9786116	3790	0.000	0.000	0.050	0.015

chr9CNV58	11941204	12175185	233981	0.018	0.000	0.067	0.025

chr10CNV59	20891019	20894574	3555	0.000	0.036	0.033	0.025

chr10CNV60	45489854	47173619	1683765	0.200	0.157	0.050	0.136

chr10CNV61	58186118	58188682	2564	0.036	0.024	0.000	0.020

chr10CNV62	58583657	58603555	19898	0.000	0.000	0.067	0.020

chr10CNV63	122759910	122774261	14351	0.127	0.000	0.000	0.035

chr10CNV64	135116379	135219995	103616	0.073	0.060	0.017	0.051

chr11CNV65	5858528	5889688	31160	0.000	0.000	0.050	0.015

chr11CNV66	21145662	21170916	25254	0.109	0.012	0.000	0.035

chr11CNV67	25574425	25580720	6295	0.036	0.012	0.000	0.015

chr11CNV68	25662734	25676867	14133	0.127	0.012	0.000	0.040

chr11CNV69	55139733	55201444	61711	0.036	0.120	0.267	0.141

chr11CNV70	55217364	55346872	129508	0.036	0.000	0.000	0.010

chr11CNV71	81182373	81192815	10442	0.018	0.120	0.117	0.091

chr11CNV72	99154024	99156143	2119	0.000	0.012	0.050	0.020

chr11CNV73	132499814	132509446	9632	0.000	0.036	0.000	0.015

chr11CNV74	134154053	134211153	57100	0.018	0.036	0.000	0.020

chr12CNV75	7888157	7982106	93949	0.018	0.000	0.050	0.020

chr12CNV76	19364102	19442103	78001	0.000	0.024	0.017	0.015

chr12CNV77	31180151	31298076	117925	0.036	0.060	0.017	0.040

chr12CNV78	62269256	62415375	146119	0.036	0.012	0.033	0.025

chr12CNV79	69162075	69162165	90	0.018	0.024	0.033	0.025

chr12CNV80	126056007	126056525	518	0.055	0.000	0.017	0.020

chr12CNV81	127794683	127830402	35719	0.000	0.012	0.017	0.010

chr12CNV82	130297674	130314013	16339	0.000	0.024	0.017	0.015

chr12CNV83	130368913	130378451	9538	0.036	0.024	0.033	0.030

chr14CNV84	43576901	43584372	7471	0.000	0.000	0.133	0.040

chr14CNV85	85358666	85376726	18060	0.018	0.024	0.000	0.015

chr14CNV86	85540054	85557089	17035	0.000	0.036	0.000	0.015

chr14CNV87	105997070	106237639	240569	0.073	0.157	0.067	0.106

chr15CNV88	30298847	30301633	2786	0.073	0.012	0.033	0.035

chr15CNV89	32530025	32587887	57862	0.127	0.072	0.067	0.086

chr15CNV90	32724681	32757729	33048	0.000	0.036	0.000	0.015

chr17CNV91	6047837	6061766	13929	0.036	0.012	0.000	0.015

chr17CNV92	14988424	14998870	10446	0.000	0.000	0.117	0.035

chr18CNV93	1915033	1964966	49933	0.018	0.036	0.000	0.020

chr18CNV94	64898548	64905367	6819	0.018	0.036	0.333	0.121

chr18CNV95	65360121	65362926	2805	0.000	0.036	0.017	0.020

chr19CNV96	15641747	15690364	48617	0.091	0.000	0.000	0.025

chr19CNV97	20423788	20473895	50107	0.109	0.084	0.033	0.076

chr19CNV98	48066441	48387680	321239	0.127	0.084	0.133	0.111

chr20CNV99	14729882	14770129	40247	0.036	0.036	0.000	0.025

chr22CNV100	17270615	17376565	105950	0.036	0.000	0.033	0.020

chr22CNV101	20718332	21554058	835726	0.218	0.253	0.167	0.217

chr22CNV102	23994408	24239811	245403	0.055	0.060	0.033	0.051

		Total	7805201				
		Average	76522				
		Median	19624				
		Minimum	90				

The 102 CNV regions identified using the 197 DNA samples obtained from 3 ethnic groups.

**Figure 2:**
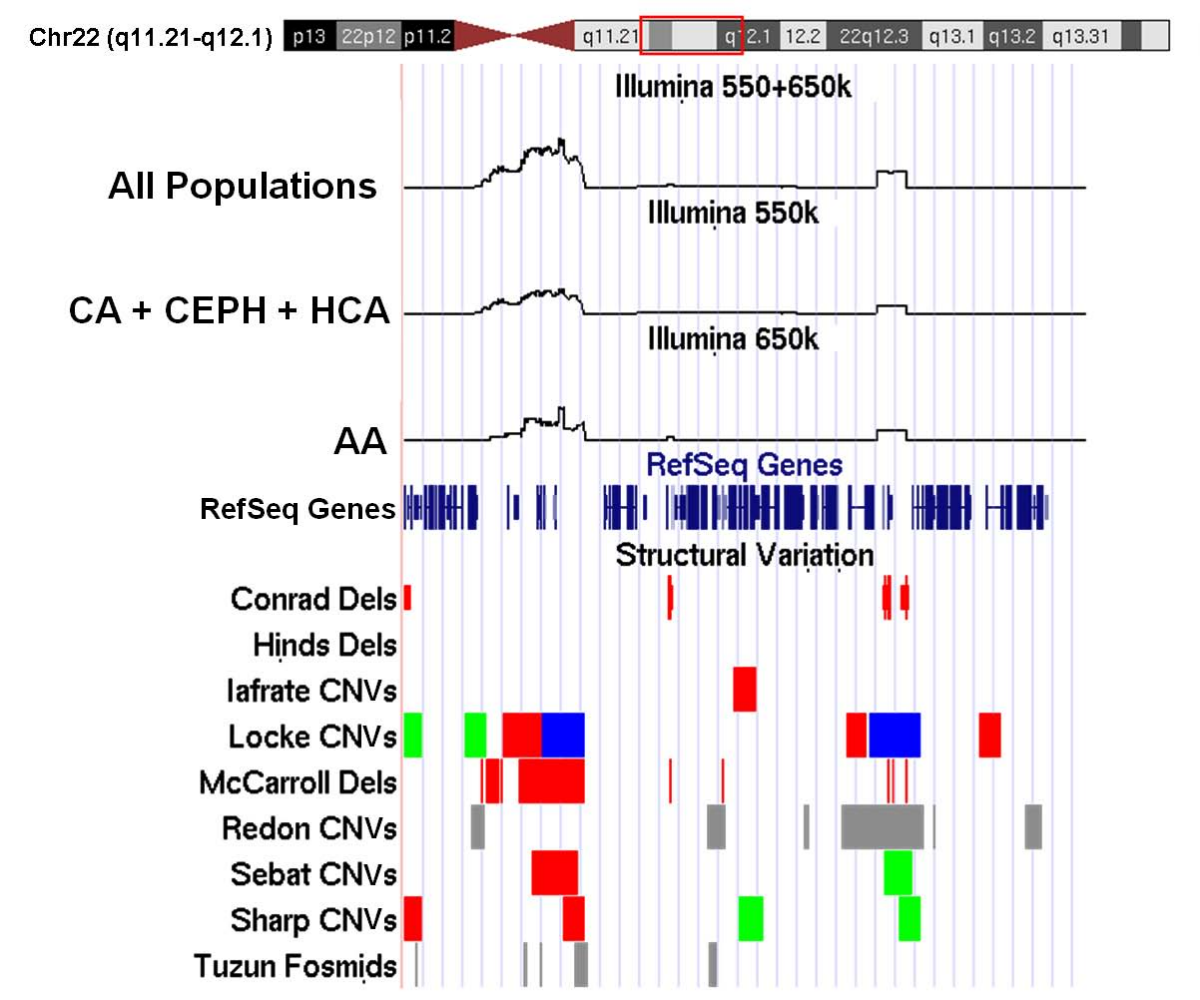
**Comparison of chromosome 22 CNV regions identified using our 197 cell line samples compared with the results of previous studies in the UCSC genome browser**. The Illumina 550 + 650 K (all samples combined), Illumina 550 K (CA, CEPH, HCA populations) and Illumina 650 K (AA samples) results in the diagram are from the present study, where spikes in the data indicate changes in CNV values. The "RefSeq Genes" row shows the locations of known genes in the human genome. In the "Structural Variation" tracks, green color indicates duplications, red indicates deletions, blue indicates both deletion and duplication, black represents an inversion and gray could be a gain or loss. "Conrad Dels" in the diagram are deletions detected by the analysis of SNP genotypes using the HapMap Phase I data, release 16c.1, CEU and YRI samples [11]. "Hinds Dels" are deletions observed during haploid hybridization analysis in 24 unrelated individuals from the Polymorphism Discovery Resource, selected for a SNP LD study [12]. "Iafrate CNVs" are from BAC microarray analysis of a population of 55 individuals [21]. "Locke CNVs" are CNV regions identified using array CGH in 269 HapMap individuals [22]. "McCarroll Dels" are deletions from genotype analysis, performed with HapMap Phase I data, release 16a [13]. "Redon CNVs" are from SNP and BAC microarray analysis of HapMap Phase II data [7]. "Sebat CNVs" represents oligonucleotide microarray analysis performed with a population of 20 normal individuals [7]. "Sharp CNVs" represents putative CNV regions detected by BAC microarray analysis in a population of 47 individuals [24]. The "Tuzun Fosmids" row consists of fosmid mapping sites detected by mapping paired-end sequences from a human fosmid DNA library [25].

We compared our loci to those in the structural variation table in the University of California Santa Cruz's (UCSC) database http://genome.ucsc.edu. Figure 2 shows an example on chromosome 22. Eighty-seven of the 102 loci that we identified overlapped with previously characterized CNVs, and 51/102 had been identified in more than one study. Of the 15 loci that had not been characterized previously, no obvious differences in size or prevalence were observed, suggesting that these are likely to be true CNVs and not the result of systematic error. In Figure 2 we have superimposed our CNV values for a portion of chromosome 22 over UCSC database data. Divergence from the baseline indicates regions of CNV, while amplitude represents prevalence. Figure 2 represents the overlap of our data (Illumina 550 + 650 K, Illumina 550 K, Illumina 650 K) at the top with previous reports at the bottom [7,11-13,21-25]. Lack of technical bias between Illumina 550 K and 650 K data is also shown since only the overlapping SNP set for the two platforms was used (Figure 2).

We also had Affymetrix U133 Plus 2.0 expression array data for the same 197 lymphoblastoid cell lines in which we assayed CNV [18], which made it possible for us to quantify the expression of genes linked to CNVs. Forty one expression array probesets mapped close to (within 500 kb) or within the 102 CNV regions that we identified. Of those 41 probesets, only 7 were expressed (17% (7/41) when compared to the 28% of the 54,000 probesets across the entire genome that were expressed) in the lymphoblastoid cell lines, with an average expression value above "100" using GCRMA normalization data (Additional file 1 Table S1).

### Gemcitabine and AraC IC_50 _value associations with CNVs

To identify gene(s) that might contribute to variation in cytidine analog-induced cytotoxicity, we next analyzed associations between CNVs and IC_50 _values for gemcitabine and AraC. We had previously performed gemcitabine and AraC cytotoxicity studies using the same cell lines, as described previously [18]. IC_50 _values for both drugs were used as phenotypes for the association studies, and the analysis was adjusted for race and gender. The association studies with gemcitabine and AraC IC_50 _value phenotypes resulted in the identification of 5 and 6 CNV regions, respectively, that showed associations with p-values < 0.05 after 1000 permutations. Although these two drugs are similar in structure, we did not observe any common CNV regions that were significantly associated with IC_50 _values for both gemcitabine and AraC. The annotation and association results for gemcitabine and AraC are listed in Tables 2 and 3, respectively.

**Table 2:** Significant associations between gemcitabine IC50 values and CNV regions.

CNV ID	Permutation P-value	Chromosome: Region	Number of SNPs	Length(bp)	SNP start-SNP end	Nearest Gene(s)
chr9CNV58	0.027	chr9:12005741-12098916	23	93176	rs10809674-rs12351590	*TYRP1*

chr1CNV5	0.031	chr1:187795066-187809352	4	14287	rs382645-rs269747	*FAM5C*

chr14CNV87	0.036	chr14:106047919-106066496	2	18578	rs4562969-rs10151262	*ADAM6*

chr11CNV74	0.042	chr11:134154053-134211153	25	57101	rs1289444-rs2155304	*B3GAT1*

chr11CNV65	0.043	chr11:5858528-5889688	12	31161	rs1377518-rs1453428	*OR52E4*

Association of CNV regions with the gemcitabine phenotype.

### CNV validation using MLPA

To experimentally validate CNVs that were significantly associated with drug cytotoxicity, we tested the 11 CNVs with permutated p-values for association that were less than 0.05 using a high-throughput method designed to quantify genomic content, multiplex ligation-dependent probe amplification (MLPA). We were unable to amplify one CNV (chr14CNV87:106047919-106066496). When we compared MLPA to CNV values on a per-sample basis, 173/197 samples matched our original QuantiSNP CNV calls. Therefore, our original analysis had a zero false positive rate for all but two regions (1.23%), and the false negative rates ranged from 0% to 65% for the 10 regions that could be amplified (Additional file 1 Table S2). The chr2CNV10 CNV, as shown in Table S2, had an exceptionally high false negative rate, and we cannot rule out the possibility that a SNP beneath the MLPA probe might be responsible.

### Functional characterization

To further characterize CNV regions, we identified genes within 500 Kb of the 11 regions that were associated with gemcitabine or AraC IC_50 _values. This relatively large region was chosen because previous studies have shown that *cis*-acting regulators can act over megabase distances [26,27]. Two of the 5 regions that were significantly associated with gemcitabine cytotoxicity contained a gene within 500 Kb of the CNV. Both regions were on chromosome 11. One chromosome 11 region, 134154053-134158019, had 25 SNPs associated with the CNV. The nearest gene, *B3GAT1*, was 372016 bp distant from the CNV (Table 2). The second chromosome 11 region, 5858528-5889688, had 12 SNPs associated with the CNV and the *OR52E4 *gene overlapped this region.

In the case of AraC, one region (divided into 3 sub-regions, as shown in Table 3) associated with AraC cytotoxicity was located on chromosome 22, with the nearest gene *LRP5L *(low density lipoprotein receptor-related protein 5-like), more than 3486 bases distant. Genes associated with chromosome 2 and 12 regions were *NPHP1 *[nephronophthisis 1 (juvenile)], *PLEKHA5 *(pleckstrin homology domain containing, family A member 5) and *GPR133 *(G protein-coupled receptor 133), respectively. *NPHP1 *and *PLEKHA5 *overlapped the CNV regions, whereas *GPR133 *was 179 Kb away from the CNV region associated with AraC. The region located on chromosome 1 overlapped the *KIF26B *gene and was 295 Kb away from a gene encoding a histone methyltransferase, *SMYD3*.

**Table 3:** Significant associations between AraC IC50 values and CNV regions.

CNV ID	Permutation P-value	Chromosome: Region	Number of SNPs	Length (bp)	SNP start-SNP end	Nearest Gene(s)
chr22CNV102	0.013	chr22:24092010-24128856	5	36847	rs713878-rs84486	*LRP5L*
	
	0.028	chr22:23999581-24091936	14	92356	rs6004527-rs713847	*LRP5L*
	
	0.028	chr22:24135224-24239811	30	104588	rs13057190-rs2780695	*LRP5L*

chr2CNV10	0.016	chr2:89714801-89874746	9	159946	rs2847840-rs842164	*FLJ40330*

chr12CNV76	0.020	chr12:19364102-19442103	16	78002	rs12825616-rs2565666	*PLEKHA5*

chr1CNV7	0.035	chr1:243707190-243713984	6	6795	rs10737772-rs12121903	*SMYD3,KIF26B*

chr2CNV11	0.044	chr2:110243431-110315618	11	72188	rs3789735-rs17463266	*NPHP1*

chr12CNV83	0.047	chr12:130368913-130378451	5	9539	rs12319995-rs4759915	*GPR133*

Association of CNV regions with the AraC phenotype.

Since we had Affymetrix U133 Plus 2.0 mRNA expression array data for the same lymphoblastoid cell lines in which we had assayed CNV [18]; we determined average expression levels for all genes associated with the CNVs. Although there were no probesets associated with *ORE52E4*, we found that the average mRNA expression levels after GCRMA normalization for probesets linked with *B3GAT1, TYRP1, FAM5C, ADAM6, LRP5L, PLEKHA5, KIF26B, NPHP1, FLJ40330*, and *GPR133 *were less than 10, suggesting either low or no expression of these genes in lymphoblastoid cells. Therefore, no further analysis was conducted with these candidates. However, one of the genes associated with AraC IC_50 _had an average expression of 247 (*SMYD3*) in our cell lines and the expression of this gene was also associated with AraC IC_50_, with a p-value of 0.0027. The Chr1CNV7 association did not pass Bonferroni correction. However, the association study was a "discovery" study - to be followed by functional genomics validation. Hence, we selected the *SMYD3 *candidate gene based on expression and possible biological relevance to cancer.

### SMYD3 functional validation

**Figure 3:**
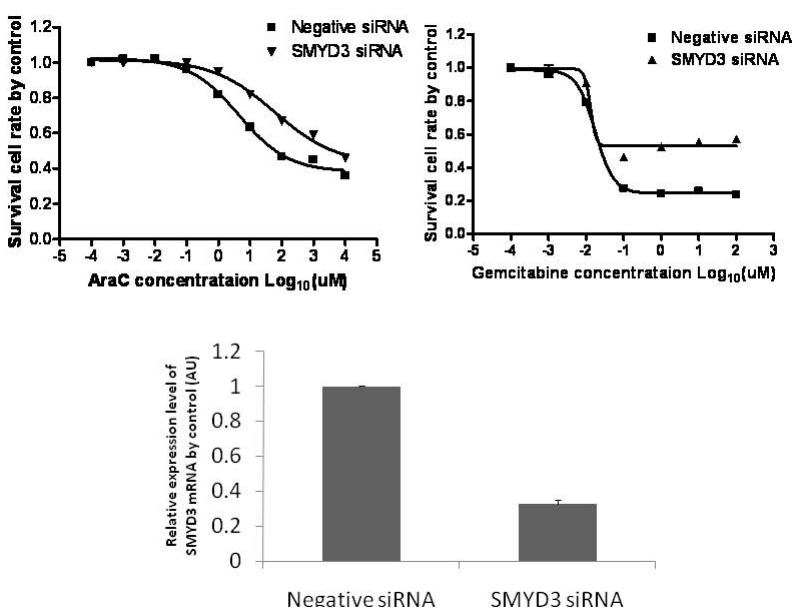
**Functional characterization for the SMYD3 candidate gene with specific siRNA knockdown**. (A) Knockdown of the SMYD3 gene in human MIApaca-2 pancreatic cancer cells resulted in increased resistance to both AraC and gemcitabine as determined by MTS assay. SEM values for 3 independent experiments were so small that they are contained within the symbols. (B) Quantitative RT-PCR for *SMYD3 *in MIApaca-2 cells. Error bars represent SEM values for three independent experiments.

It is known that knockdown of *SMYD3 *inhibits cervical carcinoma cell growth and invasion [28] and that mutations in the 5'-flanking region of *SMYD3 *may represent a risk factor for human cancer [29]. It is also known that *SMYD3 *plays crucial roles in HeLa cell proliferation and migration/invasion, so it has been suggested that it may be a useful therapeutic target in human cervical carcinomas [30]. As shown in Table 3, a CNV region located on chromosome 1 close to the *SMYD3 *gene (chr1CNV7) is associated with AraC IC_50 _value, with a permutation p-value of 0.035. The chr1CNV7 deletion occurred in 3 samples, all from Caucasian subjects, so we also tested the association in only this ethnic group. Likelihood ratio testing of linear regression of AraC log IC_50 _values against gender and storage time, with or without the relevant CNV values, gave a p-value of 0.019. In addition, analysis of IC_50 _values with the chr1CNV7 region showed that deletion of this CNV region was associated with an increase in the IC_50 _value for AraC (Table 4). To confirm results obtained from the association study, we also performed specific siRNA knockdown of the *SMYD3 *gene in human MIApaca-2 pancreatic cancer cells, followed by cytotoxicity studies. Down regulation of SMYD3 mRNA by siRNA desensitized the pancreatic cancer cells to AraC (P-value= 0.0011) when compared with cells transfected with negative control siRNA (Figure 3), a directional change consistent with the results of our CNV association study. Although, *SMYD3 *did not show a significant association with gemcitabine cytotoxicity during the association study, we also performed functional studies with that drug. We found that knockdown of the *SMYD3 *gene also made MIApaca-2 cells more resistant to gemcitabine (P-value = 0.0002) as shown in Figure 3. The Chr1CNV7 copy number was associated with gemcitabine IC50 values, (r-value = -0.01 and p-value = 0.804). While the p-value of association was insignificant, the directionality of the association was consistent with the results of the knockdown studies. In summary, knockdown of *SMYD3*, followed by cytotoxicity studies with both drugs, showed significant deviations, but the deviation was small for AraC when compared to that after gemcitabine treatment (Figure 3).

**Table 4:** AraC IC50 value mean, median and standard deviations for copy number values = 1 (deletion) and copy number value = 2 (normal).

	AraC IC_50 _values (μmol)		
**CNV Value**	**Mean**	**Median**	**Std Dev**

1	1.177	1.092	0.486
2	-0.017	-0.180	0.998

Summary of IC_50 _values for copy number = 1 and copy number = 2.

Finally, since the CNV close to the *SMYD3 *gene was significantly associated with IC_50_, and since the functional validation studies of *SMYD3 *agreed with the association study results, we expected that variation of mRNA expression for *SMYD3 *in the cell lines might be significantly correlated with IC_50_. SMYD3 mRNA expression was significantly associated with IC_50 _value, with a p-value of 0.028.

## Discussion

CNV can occur as a result of genomic rearrangements like deletion, duplication, inversion, and translocation. Features such as the presence of repetitive elements, size of the sequences, GC content, similarity and distance between the sequences play a critical role in determining susceptibility of regions to these rearrangement events [31]. Many methods have been used successfully to identify CNV regions across the genome. The high density of data from SNP platforms such as those of supplied by Illumina or Affymetrix has not only allowed us to perform genome-wide association studies to identify genotypes that are associated with a phenotype, but have also made it possible to quantify SNP alleles (log R ratios and B allele frequencies) for CNV. These log R ratios and B allele frequencies can be used to discover CNV by applying computational algorithms. However, despite advances in computational methods, the identification of intermediate sized CNVs (50 bp to 50 Kb) remains a challenge; since detection of CNVs is based on the density and spacing of probes on the platform.

In this study, we have used 550,000 SNP markers (Illumina 550 K Bead chip) to discover CNV in 197 human lymphoblastoid cell lines obtained from ethnically diverse populations. The average distance between markers on the Illumina 550 K chip is 5.8 kb, and the average size of CNVs identified in our cell lines was more than 76,000 bp, indicating that smaller CNV may be underrepresented in our study. The Illumina 550 K SNP chip was designed, in part, to interrogate gene rich regions [32]; which is an advantage with regard to a lower probability of our missing a gene-related CNV. We identified 775 CNVs in 102 regions with minor allele frequencies > 1% (Table 1). Variables such as array, coverage, intensity and CNV calling algorithms may all give different CNV calls. Therefore, we used previous copy number findings represented in the structural variation table in the UCSC database http://genome.ucsc.edu to compare with our CNVs and found that the vast majority of variant loci (87 of 102) were found in other publications, and 51/102 were represented in multiple studies. Although we did find agreement for many of our CNVs with previously reported variants, there were other CNV regions previously reported that were not identified in our study. This could be due to our stringent criteria for CNVs. It also could be due to the different platforms, methods and study populations used in different studies. In addition rare events are usually not reported.

It is known that variation in response to chemotherapy results from many factors, including gender, race, environmental factors and DNA sequence variation. DNA sequence variation may include both SNPs and CNV. Therefore, the presence of CNV is an important factor that may contribute to variation in response to chemotherapy. Specifically, the existence of CNV within or near a gene might result in differences in mRNA and protein expression. To identify possible pharmacogenomic candidate genes that might be affected by CNV, we tested the association of CNV with a drug response phenotype (IC_50_) for gemcitabine and AraC using a 197 lymphoblastoid cell line-based model system designed to make it possible to study common human genetic variation. Although tumor genome is critical for understanding response to therapy and disease pathophysiology, the germline genome is also critical, especially for drug response phenotypes. Obviously, we understand that these lymphoblastoid cell lines were EBV transformed from normal individuals, and that they were neither collected from cancer patients nor tumor tissues. Hence, we might miss some candidate genes that may be specific to cancer. However, lymphoblastoid cell lines have been shown by several groups, including ours, to be useful for identifying candidate genes or genetic variation associated with drug-induced cytotoxicity [18,33-37]. Therefore, in this study we also used these lymphoblastoid cell lines to study the possible contribution of CNVs to variation in drug response. To begin the process of understanding how variation in copy number might affect drug response phenotypes for gemcitabine and AraC, we correlated 775 CNVs with IC_50 _values in 197 lymphoblastoid cell lines. 11/102 regions were associated with gemcitabine and AraC IC_50 _values (Tables 2 and 3). We then performed MLPA to compare with and to validate the in-silico QuantiSNP CNV calls.

Since we had Affymetrix U133 Plus 2.0 expression array data for the same lymphoblastoid cell lines [18]; we determined expression levels for genes surrounding the 11 CNV regions. The *B3GAT1, LRP5L, PLEKHA5, KIF26B, NPHP1, TYRP1, FAM5C, ADAM6, FLJ40330*, and *GPR133 *genes had low expression. Only one CNV on chromosome 1 (chr1CNV7) had a gene (*SMYD3*) in close proximity that displayed high expression in the lymphoblastoid cell lines.

SMYD3, found on the q arm of chromosome 1, encodes an alternatively spliced transcript for 369 or 428 amino acids protein. Hamamoto et. al. first described SMYD3's histone methyltransferase activity with specificity for di- and tri- methylation of lys4 on Histone 3. SMYD3's histone methyltransferase activity results in transcription induction for at least 60 targets across the genome [38]. Enhanced expression of SMYD3 has been observed in numerous tumors including colorectal, hepatocellular [38] and breast cancer [39]. Overexpression experiments of SMYD3 have repeatedly shown to increase the rate of cell proliferation [38-40], while knockdown experiments result in decrease cell proliferation and cell migration while increasing apoptosis [28,41]. Our studies indicated the association of the chr1CNV7 with AraC cytotoxicity as well as correlation with SMYD3 expression. Functional validations of our results were performed with knockdown of the *SMYD3 *gene in pancreatic cancer cell lines. Knockdown made the cells more resistant to AraC, confirming the association study results, and also made them resistant to gemcitabine. Our results suggest that joining association studies with functional validation experiments may help to identify biomarkers for disease or response to therapy.

## Conclusions

We took the advantage of genome-wide SNP data obtained with 550 K Illumina Bead Chips to identify CNV regions across the genome in 197 lymphoblastoid cell lines. Association studies with gemcitabine and AraC cytotoxicity phenotypes identified CNV regions that might be associated with cytotoxicity for these two drugs. In this study we investigated the role of CNVs together with expression of neighboring genes (*B3GAT1, LRP5L, PLEKHA5, KIF26B, TYRP1, FAM5C, ADAM6, FLJ40330, NPHP1, OR52E4, GPR133 *and *SMYD3*) with drug response phenotypes. Analysis in lymphoblastoid cell lines and functional validation in cancer cell lines suggest the probable role of SMYD3 to AraC and gemcitabine drug response phenotype. The current study provides additional information with regard to the contribution of CNVs to variation in drug response for two important antineoplastic drugs and indicates that the assay of CNV should be included in pharmacogenomic studies.

## Authors' contributions

The conception of the study and interpretation of the analysis was performed conjointly by SH, KRK, CH, JPK, LL, LW and RW. Writing of the manuscript was performed by KRK, LW and SH and RW. KRK and CH performed the computational and statistical analysis, SH and LL performed the laboratory-based experiments. All of the authors read, corrected and approved the final manuscript.

## Supplementary Material

Additional file 1 **Additional file **1**, Methods Section, Table S1, Table S2** Click here for file

## References

[B1] Aklillu E Persson I Bertilsson L Johansson I Rodrigues F Ingelman-Sundberg M Frequent distribution of ultrarapid metabolizers of debrisoquine in an ethiopian population carrying duplicated and multiduplicated functional CYP2D6 alleles J Pharmacol Exp Ther 1996 278 1 441 446 8764380

[B2] Bertilsson L Dahl ML Sjoqvist F Aberg-Wistedt A Humble M Johansson I Lundqvist E Ingelman-Sundberg M Molecular basis for rational megaprescribing in ultrarapid hydroxylators of debrisoquine Lancet 1993 341 8836 63 10.1016/0140-6736(93)92546-6 8093319

[B3] Dahl ML Johansson I Bertilsson L Ingelman-Sundberg M Sjoqvist F Ultrarapid hydroxylation of debrisoquine in a Swedish population. Analysis of the molecular genetic basis J Pharmacol Exp Ther 1995 274 1 516 520 7616439

[B4] Johansson I Lundqvist E Bertilsson L Dahl ML Sjoqvist F Ingelman-Sundberg M Inherited amplification of an active gene in the cytochrome P450 CYP2D locus as a cause of ultrarapid metabolism of debrisoquine Proc Natl Acad Sci USA 1993 90 24 11825 11829 10.1073/pnas.90.24.11825 7903454 PMC48077

[B5] Roden DM Altman RB Benowitz NL Flockhart DA Giacomini KM Johnson JA Krauss RM McLeod HL Ratain MJ Relling MV Pharmacogenomics: challenges and opportunities Ann Intern Med 2006 145 10 749 757 17116919 10.7326/0003-4819-145-10-200611210-00007 PMC5006954

[B6] Dalén P Dahl ML Bernal Ruiz ML Nordin J Bertilsson L 10-Hydroxylation of nortriptyline in white persons with 0, 1, 2, 3, and 13 functional CYP2D6 genes Clin Pharmacol Ther 1998 63 4 444 452 10.1016/S0009-9236(98)90040-6 9585799

[B7] Redon R Ishikawa S Fitch KR Feuk L Perry GH Andrews TD Fiegler H Shapero MH Carson AR Chen W Global variation in copy number in the human genome Nature 2006 444 7118 444 454 10.1038/nature05329 17122850 PMC2669898

[B8] Frazer KA Ballinger DG Cox DR Hinds DA Stuve LL Gibbs RA Belmont JW Boudreau A Hardenbol P Leal SM A second generation human haplotype map of over 3.1 million SNPs Nature 2007 449 7164 851 861 10.1038/nature06258 17943122 PMC2689609

[B9] Jakobsson M Scholz SW Scheet P Gibbs JR VanLiere JM Fung HC Szpiech ZA Degnan JH Wang K Guerreiro R Genotype, haplotype and copy-number variation in worldwide human populations Nature 2008 451 7181 998 1003 10.1038/nature06742 18288195

[B10] Stranger BE Forrest MS Dunning M Ingle CE Beazley C Thorne N Redon R Bird CP de Grassi A Lee C Relative impact of nucleotide and copy number variation on gene expression phenotypes Science 2007 315 5813 848 853 10.1126/science.1136678 17289997 PMC2665772

[B11] Conrad DF Andrews TD Carter NP Hurles ME Pritchard JK A high-resolution survey of deletion polymorphism in the human genome Nat Genet 2006 38 1 75 81 10.1038/ng1697 16327808

[B12] Hinds DA Kloek AP Jen M Chen X Frazer KA Common deletions and SNPs are in linkage disequilibrium in the human genome Nat Genet 2006 38 1 82 85 10.1038/ng1695 16327809

[B13] McCarroll SA Hadnott TN Perry GH Sabeti PC Zody MC Barrett JC Dallaire S Gabriel SB Lee C Daly MJ Common deletion polymorphisms in the human genome Nat Genet 2006 38 1 86 92 10.1038/ng1696 16468122

[B14] Carter NP Methods and strategies for analyzing copy number variation using DNA microarrays Nat Genet 2007 39 7 Suppl S16 21 10.1038/ng2028 17597776 PMC2697494

[B15] Kern W Estey EH High-dose cytosine arabinoside in the treatment of acute myeloid leukemia: Review of three randomized trials Cancer 2006 107 1 116 124 10.1002/cncr.21543 16721819

[B16] Kindler HL In focus: advanced pancreatic cancer Clin Adv Hematol Oncol 2005 3 5 420 422 16167016

[B17] Wiley JS Taupin J Jamieson GP Snook M Sawyer WH Finch LR Cytosine arabinoside transport and metabolism in acute leukemias and T cell lymphoblastic lymphoma J Clin Invest 1985 75 2 632 642 10.1172/JCI111741 3871794 PMC423544

[B18] Li L Fridley B Kalari K Jenkins G Batzler A Safgren S Hildebrandt M Ames M Schaid D Wang L Gemcitabine and cytosine arabinoside cytotoxicity: association with lymphoblastoid cell expression Cancer Res 2008 68 17 7050 7058 10.1158/0008-5472.CAN-08-0405 18757419 PMC2562356

[B19] Wang K Li M Hadley D Liu R Glessner J Grant SF Hakonarson H Bucan M PennCNV: an integrated hidden Markov model designed for high-resolution copy number variation detection in whole-genome SNP genotyping data Genome Res 2007 17 11 1665 1674 10.1101/gr.6861907 17921354 PMC2045149

[B20] Colella S Yau C Taylor JM Mirza G Butler H Clouston P Bassett AS Seller A Holmes CC Ragoussis J QuantiSNP: an Objective Bayes Hidden-Markov Model to detect and accurately map copy number variation using SNP genotyping data Nucleic Acids Res 2007 35 6 2013 2025 10.1093/nar/gkm076 17341461 PMC1874617

[B21] Iafrate AJ Feuk L Rivera MN Listewnik ML Donahoe PK Qi Y Scherer SW Lee C Detection of large-scale variation in the human genome Nat Genet 2004 36 9 949 951 10.1038/ng1416 15286789

[B22] Locke DP Sharp AJ McCarroll SA McGrath SD Newman TL Cheng Z Schwartz S Albertson DG Pinkel D Altshuler DM Linkage disequilibrium and heritability of copy-number polymorphisms within duplicated regions of the human genome Am J Hum Genet 2006 79 2 275 290 10.1086/505653 16826518 PMC1559496

[B23] Sebat J Lakshmi B Troge J Alexander J Young J Lundin P Månér S Massa H Walker M Chi M Large-scale copy number polymorphism in the human genome Science 2004 305 5683 525 528 10.1126/science.1098918 15273396

[B24] Sharp AJ Locke DP McGrath SD Cheng Z Bailey JA Vallente RU Pertz LM Clark RA Schwartz S Segraves R Segmental duplications and copy-number variation in the human genome Am J Hum Genet 2005 77 1 78 88 10.1086/431652 15918152 PMC1226196

[B25] Tuzun E Sharp AJ Bailey JA Kaul R Morrison VA Pertz LM Haugen E Hayden H Albertson D Pinkel D Fine-scale structural variation of the human genome Nat Genet 2005 37 7 727 732 10.1038/ng1562 15895083

[B26] Nobrega MA Ovcharenko I Afzal V Rubin EM Scanning human gene deserts for long-range enhancers Science 2003 302 5644 413 10.1126/science.1088328 14563999

[B27] Lettice LA Horikoshi T Heaney SJ van Baren MJ van der Linde HC Breedveld GJ Joosse M Akarsu N Oostra BA Endo N Disruption of a long-range cis-acting regulator for Shh causes preaxial polydactyly Proc Natl Acad Sci USA 2002 99 11 7548 7553 10.1073/pnas.112212199 12032320 PMC124279

[B28] Wang SZ Luo XG Shen J Zou JN Lu YH Xi T Knockdown of SMYD3 by RNA interference inhibits cervical carcinoma cell growth and invasion in vitro BMB Rep 2008 41 4 294 299 18452649 10.5483/bmbrep.2008.41.4.294

[B29] Tsuge M Hamamoto R Silva FP Ohnishi Y Chayama K Kamatani N Furukawa Y Nakamura Y A variable number of tandem repeats polymorphism in an E2F-1 binding element in the 5' flanking region of SMYD3 is a risk factor for human cancers Nat Genet 2005 37 10 1104 1107 10.1038/ng1638 16155568

[B30] Silva FP Hamamoto R Kunizaki M Tsuge M Nakamura Y Furukawa Y Enhanced methyltransferase activity of SMYD3 by the cleavage of its N-terminal region in human cancer cells Oncogene 2008 27 19 2686 2692 10.1038/sj.onc.1210929 17998933

[B31] Kalari KR Casavant TL Scheetz TE A knowledge-based approach to predict intragenic deletions or duplications Bioinformatics 2008 24 18 1975 1979 10.1093/bioinformatics/btn370 18647756

[B32] Evans DM C BJ Cardon LR To what extent do scans of non-synonymous SNPs complement denser genome-wide association studies? Eur J Hum Genet 2008 16 6 718 723 10.1038/sj.ejhg.5202011 18197186

[B33] Duan S Bleibel WK Huang RS Shukla SJ Wu X Badner JA Dolan ME Mapping genes that contribute to daunorubicin-induced cytotoxicity Cancer Res 2007 67 11 5425 5433 10.1158/0008-5472.CAN-06-4431 17545624 PMC2735868

[B34] Hebbring SJ Adjei AA Baer JL Jenkins GD Zhang J Cunningham JM Schaid DJ Weinshilboum RM Thibodeau SN Human SULT1A1 gene: copy number differences and functional implications Hum Mol Genet 2007 16 5 463 470 10.1093/hmg/ddl468 17189289

[B35] Huang RS Duan S Kistner EO Bleibel WK Delaney SM Fackenthal DL Das S Dolan ME Genetic variants contributing to daunorubicin-induced cytotoxicity Cancer Res 2008 68 9 3161 3168 10.1158/0008-5472.CAN-07-6381 18451141 PMC2714371

[B36] Moyer AM Salavaggione OE Hebbring SJ Moon I Hildebrandt MA Eckloff BW Schaid DJ Wieben ED Weinshilboum RM Glutathione S-transferase T1 and M1: gene sequence variation and functional genomics Clin Cancer Res 2007 13 23 7207 7216 10.1158/1078-0432.CCR-07-0635 18056202

[B37] Welsh M Mangravite L Medina MW K T Zhang W Huang RS McLeod H Dolan ME Pharmacogenomic discovery using cell-based models Pharmacol Rev 2009 61 4 413 429 10.1124/pr.109.001461 20038569 PMC2802425

[B38] Hamamoto R Furukawa Y Morita M Iimura Y Silva FP Li M Yagyu R Nakamura Y SMYD3 encodes a histone methyltransferase involved in the proliferation of cancer cells Nat Cell Biol 2004 6 8 731 740 10.1038/ncb1151 15235609

[B39] Hamamoto R Silva FP Tsuge M Nishidate T Katagiri T Nakamura Y Furukawa Y Enhanced SMYD3 expression is essential for the growth of breast cancer cells Cancer Sci 2006 97 2 113 118 10.1111/j.1349-7006.2006.00146.x 16441421 PMC11159510

[B40] Luo XG Xi T Guo S Liu ZP Wang N Jiang Y Zhang TC Effects of SMYD3 overexpression on transformation, serum dependence, and apoptosis sensitivity in NIH3T3 cells UBMB Life 2009 61 6 679 684 10.1002/iub.216 19472189

[B41] Zou JN Wang SZ Yang JS Luo XG Xie JH Xi T Knockdown of SMYD3 by RNA interference down-regulates c-Met expression and inhibits cells migration and invasion induced by HGF Cancer Letters 2009 280 1 78 85 10.1016/j.canlet.2009.02.015 19321255

