# Supplementary material for: Copy number variation and cytidine analogue cytotoxicity: A genome-wide association approach
Source: BMC Genomics. 2010 Jun 4;11:357. doi: 10.1186/1471-2164-11-357 (PMC2894803; doi:10.1186/1471-2164-11-357)
Supplement: Additional file 1 — , Methods Section, Table S1, Table S2 [file 1471-2164-11-357-S1.DOC]

**Additional file 1 Methods Section**

Segregation Algorithm to identify CNVs

---------------------------------------------------

After obtaining raw CNV values from the quantiSNP algorithm, we converted the values as outlined subsequently.

The transformation

if CN<2 then new-CN=1

if CN>2 then new-CN=3

if CN=2 then new-CN=2

was applied.

SNPs from the same region that shared the same transformed CN were merged. For example, if we had a region with 3 SNPs, entries in the table below represent copy number:

Cell Line 1 Cell Line 2 Cell Line 99 Cell Line 100

SNP1 2 2 2 1

SNP2 2 2 2 1

SNP3 1 2 2 1

Check whether all entries in row 1 equal those in row 2. If positive, then merge the SNPs, otherwise do not do anything. Using this procedure, the table above would create 2 groups of SNPs, SNP1 and SNP2 would be in one group, and SNP 3 would be the other group. In this way, all resulting groups of SNPs can be identified by a unique ‘CNVregionID’.

**Additional file 1 Table S1**

The mRNA expression of probesets surrounding the CNV regions identified in this study.

| ProbeSet | CNV_ID | Gene Symbol | Build | Avg. Exp | Median Exp |
| --- | --- | --- | --- | --- | --- |
| 228758_at | chr3CNV24 | BCL6 | 36.1 | 6.33627 | 6.142 |
| 243874_at | chr3CNV24 | --- | 36.1 | 6.60097 | 6.401 |
| 239249_at | chr3CNV24 | --- | 36.1 | 9.0058 | 8.936 |
| 239706_x_at | chr19CNV98 | --- | 36.1 | 14.0575 | 13.5 |
| **218788_s_at** | **chr1CNV7** | **SMYD3** | **36.1** | **247.024** | **234.1** |
| 202821_s_at | chr3CNV24 | LPP | 36.1 | 5.84835 | 5.533 |
| 210196_s_at | chr19CNV98 | PSG1 | 36.1 | 10.0539 | 9.528 |
| 239583_x_at | chr19CNV98 | PLAC7 | 36.1 | 15.0586 | 14.39 |
| 237780_at | chr3CNV24 | --- | 36.1 | 5.03011 | 4.932 |
| 237483_at | chr12CNV76 | --- | 36.1 | 9.7152 | 9.287 |
| 220952_s_at | chr12CNV76 | PLEKHA5 | 36.1 | 5.40806 | 5.053 |
| 216207_x_at | chr2CNV10 | IGKC | 36.1 | 5078.14 | 3995 |
| 205602_x_at | chr19CNV98 | PSG7 | 36.1 | 7.91271 | 7.579 |
| 233824_at | chr3CNV24 | --- | 36.1 | 6.95479 | 6.634 |
| 209738_x_at | chr19CNV98 | PSG6 | 36.1 | 5.75052 | 5.608 |
| 236773_at | chr20CNV99 | --- | 36.1 | 41.2937 | 34.01 |
| 220301_at | chr18CNV94 | CCDC102B | 36.1 | 7.47199 | 7.286 |
| 219250_s_at | chr20CNV99 | FLRT3 | 36.1 | 13.8736 | 6.941 |
| 235278_at | chr20CNV99 | MACROD2 | 36.1 | 2529.42 | 2289 |
| 243573_at | chr3CNV24 | --- | 36.1 | 6.70794 | 6.469 |
| 242468_at | chr20CNV99 | --- | 36.1 | 34.5363 | 23.33 |
| 222853_at | chr20CNV99 | FLRT3 | 36.1 | 9.80145 | 4.697 |
| 220002_at | chr1CNV7 | KIF26B | 36.1 | 19.2706 | 15.75 |
| 215176_x_at | chr2CNV10 | LOC100130100 | 36.1 | 5250.11 | 4160 |
| 214902_x_at | chr3CNV24 | --- | 36.1 | 77.5794 | 74.69 |
| 217336_at | chr20CNV99 | RPS10 | 36.1 | 146.724 | 142.4 |
| 214989_x_at | chr12CNV76 | --- | 36.1 | 169.303 | 163.2 |
| 216353_s_at | chr20CNV99 | --- | 36.1 | 8.12114 | 7.782 |
| 238617_at | chr1CNV7 | --- | 36.1 | 15.1642 | 12.99 |
| 202822_at | chr3CNV24 | LPP | 36.1 | 5.43435 | 5.034 |
| 213005_s_at | chr9CNV54 | KANK1 | 36.1 | 14.8611 | 5.107 |
| 216596_at | chr15CNV88 | DKFZP434L187 | 36.1 | 8.83679 | 8.381 |
| 236439_at | chr3CNV24 | --- | 36.1 | 6.90048 | 6.743 |
| 232010_at | chr4CNV31 | FSTL5 | 36.1 | 6.50955 | 5.749 |
| 210123_s_at | chr15CNV88 | CHRFAM7A | 36.1 | 7.82405 | 6.995 |
| 203140_at | chr3CNV24 | BCL6 | 36.1 | 6.16624 | 5.361 |
| 230861_at | chr15CNV88 | DKFZP434L187 | 36.1 | 5.23857 | 5.106 |
| 230996_at | chr3CNV24 | LPP | 36.1 | 4.83771 | 4.747 |
| 210705_s_at | chr11CNV65 | TRIM5 | 36.1 | 682.357 | 641.4 |
| 237909_at | chr14CNV87 | ADAM6 | 36.1 | 4.936 | 9.466 |
| 205694_at | chr9CNV58 | TYRP1 | 36.1 | 4.37 | 4.58 |
| 217562_at | chr1CNV5 | FAM5C | 36.1 | 6.01 | 6.55 |
| 215990_s_at | chr3CNV24 | BCL6 | 36.1 | 4.77579 | 4.689 |
| 233040_at | chr12CNV76 | PLEKHA5 | 36.1 | 7.67713 | 7.406 |

**Additional file 1 Table S2**

True negative, true positive, false negative and false positive rates for 10 CNV regions compared with results obtained with the MLPA assay.

| Region | True  Negative | True  Positive | False  Negative | False  Positive | N |
| --- | --- | --- | --- | --- | --- |
| chr11CNV65 | 183 | 3 | 1 | 0 | 187 |
| chr11CNV74 | 159 | 2 | 0 | 2 | 163 |
| chr12CNV76 | 184 | 3 | 0 | 0 | 187 |
| chr12CNV83 | 180 | 4 | 2 | 0 | 186 |
| chr1CNV5 | 158 | 2 | 3 | 0 | 163 |
| chr1CNV7 | 182 | 3 | 2 | 0 | 187 |
| chr22CNV102 | 155 | 8 | 0 | 0 | 163 |
| chr2CNV10 | 166 | 7 | 13 | 0 | 186 |
| chr2CNV11 | 156 | 4 | 1 | 2 | 163 |
| chr9CNV58 | 160 | 3 | 0 | 0 | 163 |
